# Uterine rupture during induction of labor at 15 + 2 weeks’ gestation: A case report

**DOI:** 10.1097/MD.0000000000042144

**Published:** 2025-04-18

**Authors:** Shaou Wang, Xiaoyan Zhu, Jianhao Sun, Jiajia Lu

**Affiliations:** a Department of Ultrasound, The First People’s Hospital of Xiaoshan District, Xiaoshan Affiliated Hospital of Wenzhou Medical University, Hangzhou, China.

**Keywords:** induced labor in mid-pregnancy, scarred pregnancy, uterine rupture

## Abstract

**Rationale:**

Uterine rupture is a rare obstetric complication that can quickly and directly threaten the lives of mothers and babies.

**Patient concerns:**

We report a case of a patient with 15w + 2d gestation scarred pregnancy requesting induction of labor, due to insufficient preoperative evaluation and increased dose of intraoperative induction medication, uterine rupture occurred due to tonicity of uterine contractions.

**Diagnoses:**

Uterine rupture due to mid-pregnancy induction of labor in a patient with scarred pregnancy.

**Interventions:**

The patient underwent surgical treatment, removal of the embryo and uterine suturing.

**Outcomes:**

The patient recovered well and was discharged. Follow up visit to our hospital for review, uterus recovered well.

**Lessons:**

We should improve the preoperative evaluation of patients with scarred pregnancy, comprehensively consider the choice of induction method, the control of medication dosage, strengthen the intraoperative monitoring of high-risk patients, and deal with problems in a timely manner in an effort to minimize the risk of uterine rupture and harm.

## 1. Introduction

Uterine rupture is the splitting of the body or lower part of the uterus during pregnancy or labor. Depending on the degree of rupture it can be categorized as complete uterine rupture or incomplete uterine rupture. Complete ruptures are usually more serious and potentially life-threatening to both the fetus and the mother.^[1]^ World Health Organization (WHO) statistics on the incidence of uterine rupture around the world range from 0.016% to 0.3%.,^[2]^ Although uterine rupture is a rare obstetric complication, its outcome can be catastrophic. hen it occurs, uterine rupture is a rapid and immediate threat to the life of both mother and baby.^[3,4]^

## 2. Case presentation

Patient, female, 33 years old, complained of a 6-year history of hypertension, with good blood pressure control on weekdays, denied history of other diseases, and 2 previous cesarean sections.

The patient was diagnosed with “early intrauterine pregnancy” 2 months ago in an outside hospital, and her blood pressure was monitored at that time: 160/105 mm Hg; She was given labetalol tablets 1#bid oral symptomatic treatment, and her blood pressure was poorly controlled. Two months later, the pregnant woman and her family asked for termination of the pregnancy, and she was admitted to our hospital.

After admission, the relevant examinations were perfected: pulse 90 times/min, breathing 18 times/min, blood pressure 128/86 mm Hg, body temperature 36.6°C. 24-hour ambulatory blood pressure: average blood pressure: 137/85 mm Hg. Ultrasound examination: fetal head position, double top diameter 3.0 cm, femur diameter 1.4 cm, fetal heart rate and fetal movement, admission diagnosis: second-trim abortion; pregnancy with hypertension; and pregnancy with scarred uterus.

**Figure 1.**
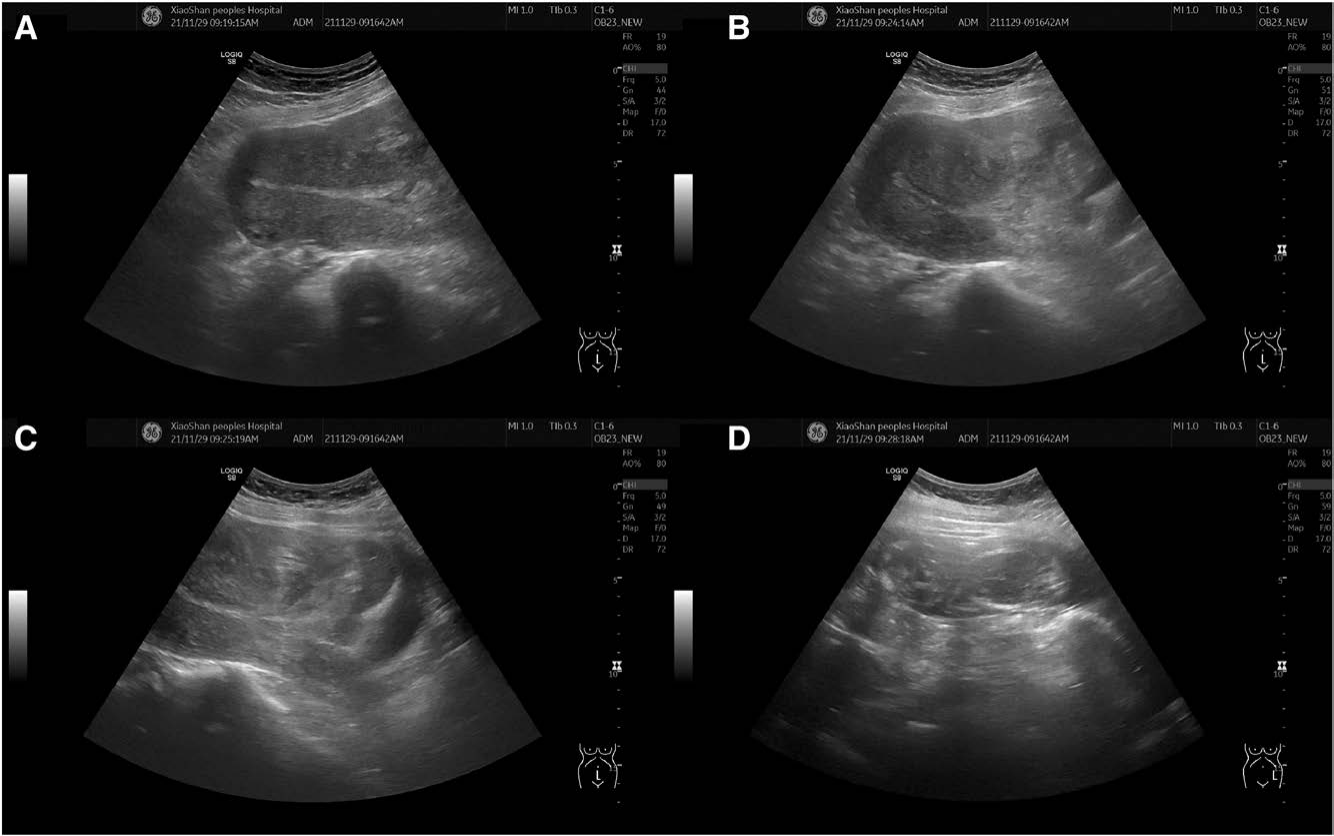
Ultrasonography of uterine rupture during induction of labor in a pregnant woman at 15 + 2 weeks of gestation. A. No visible fetal echoes in the uterine cavity. B. Disruption of myometrial continuity in the lower segment of the anterior wall. C. Mixed clot of placenta seen protruding from the uterine cavity into the abdominal cavity at the incision in the lower part of the anterior wall. D. An amniotic sac-encapsulated fetal echo is seen in the abdominal cavity.

On the second day of admission, oral mifepristonum tablets was taken 3 times, 2 tablets each time, and the cervix was softened once every 12 hours, during which the patient had no abdominal pain and no vaginal bleeding. Oral misoprostol tablets 600 µg on an empty stomach at 7 am on the third day, 2 hours later, the patient had contractions and irregular abdominal pain, no vaginal bleeding and pregnancy tissue discharge, 4 hours later the patient still did not have pregnancy tissue discharge, abdominal pain, weak intensity, then gave carboprost methylate suppositorites 1mg to soften the cervix and promote uterine contractions, and at 9 o’clock in the morning on the fourth day, there was still no fetal discharge, but the patient say that the pain began to be unbearable since the night. Physical examination: local tenderness on the side of the lower abdominal uterine body, gynecological examination after disinfection: a small amount of bloody discharge from the vagina, closed uterine opening, no obvious blood outflow, only mucus, considering the poor efficacy of misoprostol and card pregnancy thrombosis on the patient, and the history of 2 cesarean sections, the possibility of uterine rupture is not excluded, and ultrasound examination, ultrasound shows: uterus enlargement, no obvious fetal echo in the uterine cavity (Fig. 1A), The muscular echo at the incision of the lower segment of the anterior wall is interrupted, and the uneven echo is visible locally extending outward from the uterine cavity, and the boundary seems to be visible (Fig. 1B and C), A deformed fetal echo can be seen in the left abdomen (Fig. 1D), a small amount of liquid dark area is seen around it, and no obvious liquid dark area is seen in the abdomen-pelvic cavity, and ultrasound shows that uterine rupture is considered first.

**Figure 2.**
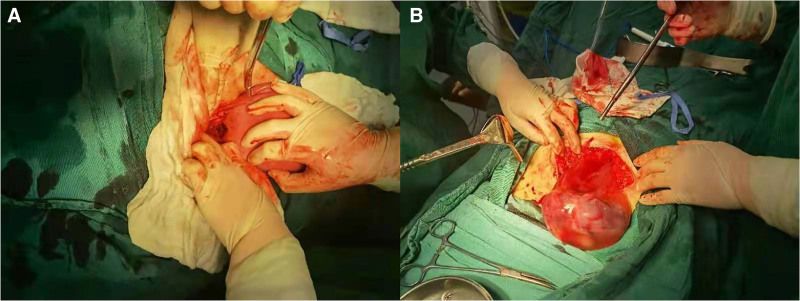
Intraoperative picture of uterine rupture during induction of labor in a pregnant woman at 15 + 2 weeks’ gestation. A. Rupture of the lower part of the anterior wall of the uterus. B. Fetus encapsulated by amniotic sac protruding into the abdominal cavity uterus.

Emergency surgery, intraoperatively, the patient showed hypertrophy of the abdominal fat layer and anatomical disorganization of the tendon sheath with uneven thickness of the tendon sheath layer (self-reported history of previous incision infection with secondary suturing). Total loss of muscle layer, the uterine exploratory incision was split by 5 cm (Fig. 2A). Poorly localized borders in the lower segment of the philtrum, A small amount of blood is visible in the pelvis. The membranes encircle the dark purple placenta and the amniotic sac wraps around the stillborn limb to form a closed whole. (Fig. 2B), Border irregularities, Partially outside the incision and on the left side of the uterus, Partially in the uterine cavity, Saline gauze pushed through the intestinal tube, The complete removal of the placenta and dead fetus after protecting the incision. Careful examination of the uterus by holding it out of the incision did not reveal a hematoma of the broad ligament or any other laceration. The lower uterine fissure was then repaired, and the myometrium and the plasma membrane layers of the fissure were closed in 2 layers using 1/0 absorbable sutures and continuous suturing to ensure solid closure of both layers of the fissure. Intraoperative scrutiny showed no active bleeding, and bleeding was well controlled after repair. Then, the abdominal cavity was thoroughly cleaned to confirm that there were no residual foreign bodies, and gauze and instruments were counted to ensure accuracy. Finally, the abdomen was closed layer by layer and closed at the skin level using internal suture technique to ensure the surgical area was airtight and to minimize the risk of infection. The procedure went well. Intraoperative bleeding of 30 mL. Blood pressure is stable. Postoperative diagnosis: G3P2 pregnancy 15w + 2d intrauterine pregnancy induced at mid-pregnancy, uterine rupture, gestational hypertension, and gestational uterine scarring.

## 3. Discussion

The incidence of uterine rupture has been on the rise in recent years.^[5,6]^ It as a serious complication of obstetrics. Which when it occurs can result in serious maternal and fetal outcomes such as severe hemorrhage, disseminated intravascular coagulation, fetal distress, and intrauterine fetal death. Even directly endangering maternal life. The typical clinical presentation is abdominal pain, Abnormal fetal heartbeat, vaginal bleeding, and in some people, nausea, vomiting, hematuria, and decreased blood pressure. Symptoms can vary depending on the cause of the rupture. A large number of scholars at home and abroad have done retrospective studies on uterine rupture. A number of causes of uterine rupture have been found, including cesarean section, history of uterine manipulation, medically induced or induced labor, placental implantation, uterine anomalies, and laparoscopic surgery. One of the most common causes of uterine rupture is scarred uterus due to cesarean section.^[7–10]^ Also during induced labor, Inappropriate use or abuse of prostaglandin analogs is also a common cause of uterine rupture.^[11–13]^

This patient had a history of 2 cesarean deliveries and a history of hypertension. In Tahseen^[14]^ the risk of uterine rupture was noted in the published literature to be significantly higher in pregnant women with a history of a second cesarean section than in those with a history of a primary cesarean section.Therefore, this patient was at high risk for uterine rupture. However, the preoperative ultrasonography did not assess the thickness, continuity, presence of diverticula, or integrity of the plasma layer of the maternal scar. The preoperative assessment was inadequate. During abortion, mifepristone was used to soften the cervix and promote cervical canal ripening, followed by 600 μg of misoprostol to promote contractions, a dose that is high in patients with scarred uteri. In 2022, the WHO released the updated abortion care guideline.About the second trimester top regimen, the WHO recommended the use of 200 mg of mifepristonum, followed 24 to 48 hours later by 400 μg of misoprostol administered every 3 hours buccally, sublingually or vaginally.^[15]^ Six hours after the oral administration of misoprostol, the patient still had no expulsion of pregnancy tissue and had only irregular abdominal pain of weak intensity, and a 1 mg cargometrine pessary was inserted intravaginally to soften the cervix and promote contractions. This was an inappropriate use of medication. In patients with scarred uteri, the use of multiple medications should be avoided, as the use of a combination of suppositories may lead to overly strong contractions, increasing the risk of uterine rupture.^[16]^ Therefore, the cause of uterine rupture in this case can be analyzed as strong contractions – weak scarring – cervical immaturity.

In summary, this case highlights the importance of comprehensive assessment and individualized management of abortion in patients with uterine scarred pregnancy. For patients with scarred uterus, scarring should be assessed in detail by ultrasound in the early stages of pregnancy, and the principle of standardized medication should be strictly followed during induction of abortion to avoid superimposed use of multiple medications. In addition, because early symptoms of uterine rupture are not specific,^[17]^ dynamic monitoring should be strengthened, especially in the presence of abdominal pain or abnormal fetal heartbeat, and timely ultrasound examination should be performed to exclude the possibility of uterine rupture.

## Author contributions

**Conceptualization:** Xiaoyan Zhu, Jianhao Sun.

**Methodology:** Jiajia Lu.

**Writing – original draft:** Shaou Wang.
